# Ion Selectivity and Stability Enhancement of SPEEK/Lignin Membrane for Vanadium Redox Flow Battery: The Degree of Sulfonation Effect

**DOI:** 10.3389/fchem.2018.00549

**Published:** 2018-11-12

**Authors:** Jiaye Ye, Xuechun Lou, Chun Wu, Sujuan Wu, Mei Ding, Lidong Sun, Chuankun Jia

**Affiliations:** ^1^College of Materials Science and Engineering, Changsha University of Science and Technology, Changsha, China; ^2^State Key Laboratory of Mechanical Transmission, School of Materials Science and Engineering, Chongqing University, Chongqing, China; ^3^National Engineering Laboratory of Highway Maintenance Technology, Changsha University of Science & Technology, Changsha, Hunan, China

**Keywords:** vanadium flow battery, Sulfonated poly(ether ether ketone), SPEEK/lignin composite membrane, ion selectivity, degree of sulfonation

## Abstract

A membrane of high ion selectivity, high stability, and low cost is desirable for vanadium redox flow battery (VRB). In this study, a composite membrane is formed by blending the sulfonated poly (ether ether ketone) with lignin (SPEEK/lignin), and optimized by tailoring the degree of sulfonation. The incorporation of lignin into the SPEEK matrix provides more proton transport pathway and meanwhile adjusts the water channel to repulse vanadium ions. The VRB cells assembled with the composite membranes exhibit high coulombic efficiency (~99.27%) and impressive energy efficiency (~82.75%). The cells maintain a discharge capacity of ~95% after 100 cycles and ~85% after 200 cycles at 120 mA cm^−2^, much higher than the commercial Nafion 212. The SPEEK/lignin composite membranes are promising for application in VRB system.

## Introduction

The vanadium redox flow battery (VRB) has attracted tremendous interest as a large-scale energy storage technique, for environment protection and sustainable development, in light of its long cycle life, fast response, flexible design, and great reliability via a cost-effective and eco-friendly means (Zhang et al., 2014a; Jia et al., 2015b; Yang et al., 2015; Ye et al., 2016; Lu et al., 2017; Wu et al., 2018, 2019). Proton exchange membrane is a key component in the flow battery, which performs as a separator to isolate the positive and negative electrolyte compartments, and meanwhile to conduct protons (Jia et al., 2012; Yu et al., 2016). An ideal membrane is expected to exhibit high proton conductivity, good chemical and mechanical stability, accurate ion selectivity, and low-cost fabrication approach (Jia et al., 2010; Ding et al., 2018; Yuan et al., 2018). To date, the commercial Nafion membranes have been widely used in VRBs, because of its good proton conductivity, remarkable chemical and mechanical stability (Li et al., 2014a; Dai et al., 2017). However, the high crossover rate of vanadium ions hampers its further application in VRBs (Zhang et al., 2018). Accordingly, several groups have been devoted to enhancing the performance of Nafion membranes by different methods, such as changing the casting solvent and annealing temperature (Dai et al., 2017), altering pretreatment process (Jiang et al., 2016), employing surface modification (Teng et al., 2015), forming composite structure with organic materials, inorganic materials or both (Zeng et al., 2008; Mai et al., 2011; Teng et al., 2013, 2014). The modified membranes usually lower the permeability of vanadium ions. Nevertheless, the extremely high cost of the Nafion membranes is a critical barrier for VRB commercialization (Yuan et al., 2017). Therefore, it is appealing to explore alternative systems of high ion selectivity, good stability and low cost toward practical application.

The sulfonated hydrocarbon polymers and their derivatives are promising candidates as the substitutional membranes (Wang et al., 2013). The sulfonated poly (ether ether ketone; SPEEK) is of particular interest, in view of its low vanadium ion permeability, simple preparation, high chemical, and mechanical stability (Winardi et al., 2014; Jia et al., 2015a). More importantly, the SPEEK membrane is cost-effective, only accounting for about several tenths of the commercial Nafion (DuPont). To enhance the proton conductivity, organic or inorganic materials with abundant hydrophilic groups are usually introduced to form composite membranes. Moreover, the interaction between the additives and the SPEEK matrix also ensures a good chemical and mechanical stability under harsh condition during the VRB operation. Jia et al. (2015a) prepared a composite membrane by blending the SPEEK with functionalized carbon nanotubes. The membrane shows not only high coulombic efficiency (CE), voltage efficiency (VE), and energy efficiency (EE), but also good mechanical stability and low capacity loss, compared with the pristine SPEEK and Nafion 212 membrane. Other SPEEK-based composite membranes also exhibited excellent performances for VRB application, such as SPEEK/SPES [sulfonated poly (ether sulfone)] (Ling et al., 2012), SPEEK/GO (graphene oxide; Kong et al., 2016; Park and Kim, 2016), SPEEK/QPEI [quaternized poly(ether imide)] membranes (Liu et al., 2014, 2015).

**Figure 1:**
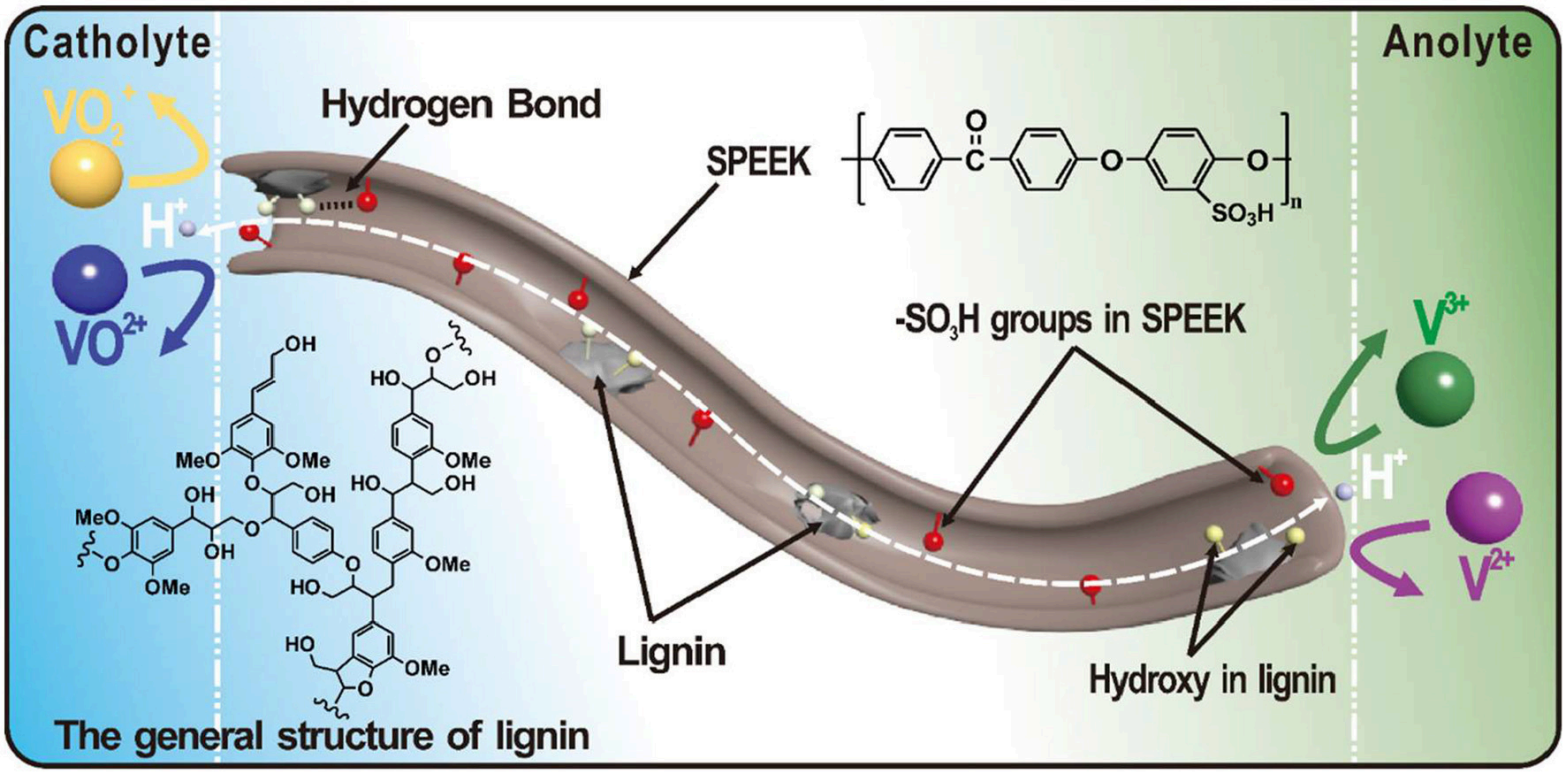
The general structure of lignin and schematic illustration showing the water channel in a composite membrane for vanadium redox flow battery.

Considering the cost of the additives, the lignin has recently been focused in our group, which is a byproduct in paper industry and bio-fuel producing process (Gong, 2016). The lignin possesses abundant hydroxyl groups and thus improves the wettability of the polymer matrix and promotes the proton conductivity of the blend membrane. Figure 1 shows a general structure of lignin, in which the phenol propane unit is linked by alkyl–aryl, alkyl–alkyl and aryl–aryl ether bonds (Tolba et al., 2010; Ge et al., 2014; Zhang et al., 2014b; Zhu et al., 2016; Atifi et al., 2017; Rahman et al., 2018). The lignin interlaces with the SPEEK substrate and reduces the size of water channels. This gives rise to enhanced proton conductivity and meanwhile suppressed ion permeability. On the other hand, the degree of sulfonation (DS) in the SPEEK matrix, i.e., the amount of -SO_3_H groups, also affects the proton conductivity. In general, the conductivity increases with the DS. However, a high DS seriously influence the chemical and mechanical stability of the membrane (Xi et al., 2015). In this study, the DS effect on the ion selectivity and stability was systematically studied. An optimized degree of sulfonation was proposed toward the application in VRBs.

## Experimental

### Materials and membranes preparation

The lignin powders (Sigma-Aldrich) were soaked in 2 M hydrochloric acid solution (mass to volume ratio, 1 g per 20 mL) and stirred for 2 h. After that, sodium hydroxide solution was used to neutralize the above mixture, followed by filtration and freeze drying. The DS of SPEEK was controlled to about 41, 50, 59, and 72%, which were measured by titration method according to our previous work (Jia et al., 2015a). For the preparation of SPEEK/lignin composite membranes, 1.6 g of SPEEK was dissolved in 50 mL dimethyl sulfoxide (DMSO), and stirred at 60°C until being dissolving completely. After cooling down to room temperature, 240 mg pretreatment lignin powder was added and vigorously stirred overnight. The mixture was casted on a home-made glass plate and dried in oven at 100°C for solvent evaporation. After cooling, the membrane was peeled off from the glass and soaked in water immediately. Based on the mass ratio of lignin to SPEEK, the composite membranes were named as SPEEK41/L15, SPEEK50/L15, SPEEK59/L15, and SPEEK72/L15. Commercial Nafion 212 membrane (DuPont) was used as the reference. All of the reagents were used as received.

### Characterization

Fourier transform infrared spectroscopy (FT-IR, Thermo Fisher Nicolet iS10) was investigated in the range of 400–4,000 cm^−1^. The microstructure of the as-prepared membranes was examined with field-emission scanning electron microscopy (FESEM, Zeiss Auriga FIB/SEM). Water uptake (WU) was measured as follow: (1) dry membrane was soaked into distill water for 24 h; (2) the membrane was taken out and cleaned with filter paper immediately; (3) the cleaned membrane was weight by Mettler-Toledo analytical balance (ME204E). Swelling ratio (SR) was obtained by measuring the length variation of the membrane before and after immersing in deionized water for 24 h. The WU and SR of these membranes can be calculated through the following equations (Ling et al., 2012).

(1) $$\begin{array}{r} \text{W}U=\frac{{\text{W}}_{\text{wet}}-{\text{W}}_{\text{dry}}}{{\text{W}}_{\text{dry}}} \end{array}$$

(2) $$\begin{array}{r} \text{SR}=\frac{{\text{L}}_{\text{wet}}-{\text{L}}_{\text{dry}}}{{\text{L}}_{\text{dry}}} \end{array}$$

where the *W*_dry_ and *W*_wet_ are the weight of the membrane before and after soaking, respectively; the *L*_dry_ and *L*_wet_ are the length of the membrane before and after soaking, respectively.

**Figure 2:**
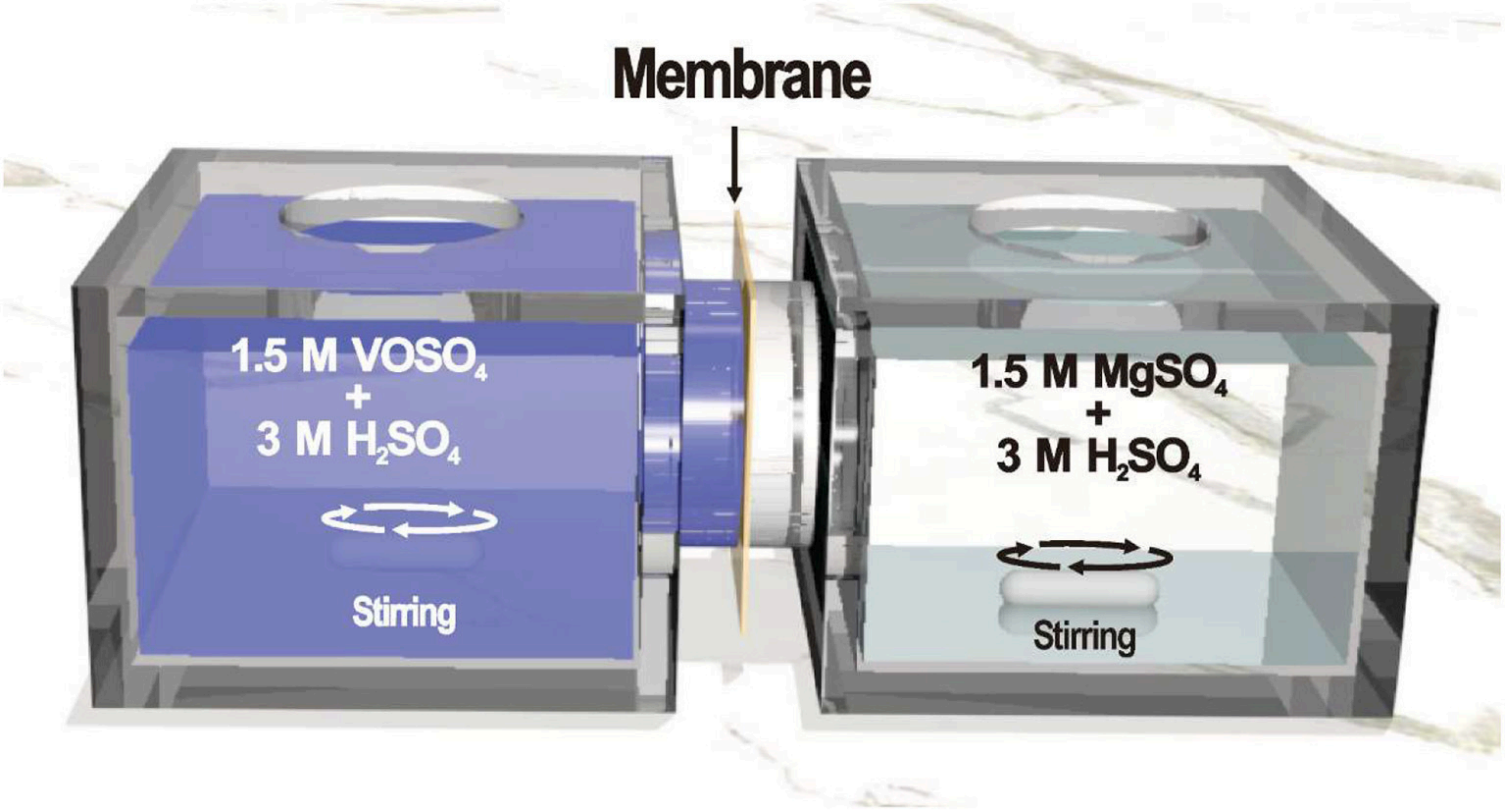
A typical diffusion cell to measure VO^2+^ permeability.

The permeability of VO^2+^ ion across the membranes was investigated as follow: (1) isolating two reservoirs that were filled 70 mL of 1.5 M VOSO_4_ in 3.0 M H_2_SO_4_ solution and 70 mL of 1.5 M MgSO_4_ in 3.0 M H_2_SO_4_, respectively, with a membrane of 2.01 cm^2^ active area; (2) stirring continuously and measuring the concentration of VO^2+^ in MgSO_4_ compartment at 24 h intervals by TU-1900 UV-vis spectrometer; (3) sampling with replacement to keep the solution volume stable. A typical experimental setup was shown in Figure 2. The permeability value of the membrane can be calculated using Equation (3) (Jia et al., 2015a):

(3) $$\begin{array}{r} \text{V}\frac{\text{d}\text{C}\left(\text{t}\right)}{d\text{t}}=\text{A}\frac{\text{P}}{\text{L}}\left(\text{C}-\text{C}\left(\text{t}\right)\right) \end{array}$$

where V, A, *P, L*, C, and *C*(*t*) are the volume of the VOSO_4_ solution, the effective area of the membrane, the permeability of the vanadium ions, the thickness of the membrane, the initial concentration of VO^2+^ in the VOSO_4_ compartment, and the vanadium concentration in the MgSO_4_ compartment at the moment *t*, respectively.

The area resistance (*R*) of membranes was investigated by a resistance tester (DME-20, DM, China). The electrolytes in both compartments were 1.5 M VOSO_4_ and 3 M H_2_SO_4_, and the conductivity (σ) of the membrane can be calculated as follow (Ling et al., 2012):

(4) $$\begin{array}{r} \sigma =\frac{\text{L}}{\text{AR}} \end{array}$$

where *R* represents the resistance difference between the cell with and without membrane, *L* is the thickness of the membrane, and *A* is the active area of membrane (13.5 cm^2^).

### Cell testing

The VRB single cell consisted of a composite membrane (13.5 cm^2^) sandwiched between two carbon felt electrodes (13.5 cm^2^), and two graphite polar plates (current collectors). The 1.5 M VO${}_{2}^{+}$ in 3.0 M H_2_SO_4_ and 1.5 M V^3+^ in 3.0 M H_2_SO_4_ solutions were used as catholyte and anolyte, respectively. The cell performance was measured by Arbin battery testing system (BT-I, Arbin, USA) including open circuit voltage decay (OCV), long cycle charge-discharge, and rate performance. The OCV (75% state of charge) was terminated when the voltage of the testing cell declined below 0.85 V. The potential range was between 0.7 and 1.75 V at room temperature.

## Results and discussion

### Membrane characterization

**Figure 3:**
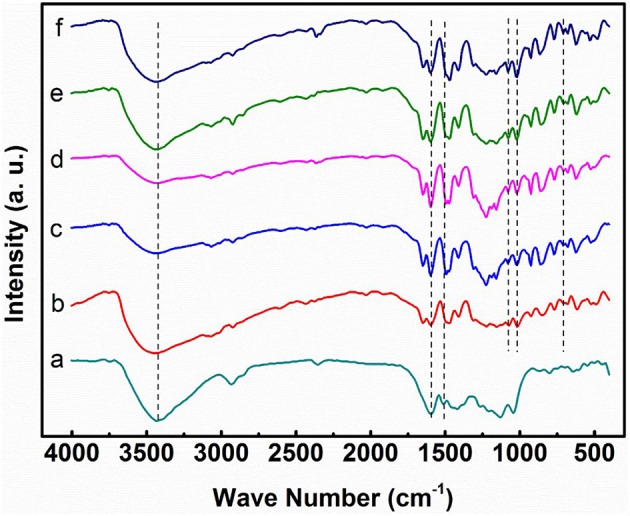
FT-IR spectra of **(A)** lignin powder, **(B)** pure SPEEK membrane, **(C)** SPEEK41/L15, **(D)** SPEEK50/L15, **(E)** SPEEK59/L15, **(F)** SPEEK72/L15.

Figure 3 shows the FT-IR spectra of lignin, pure SPEEK, and the composite membranes. The broad band at 3,400 cm^−1^ is ascribed to the hydrogen bond and OH vibration. For lignin, the peaks at 2,938 and 2,849 cm^−1^ are assigned to the C-H stretch in methyl and methylene group (-CH_2_-), and those at 1,594 and 1,511 cm^−1^ are attributed to the aromatic rings of phenyl propane skeleton (characteristic bands of lignin; Faix, 1991). For SPEEK membrane alone, the following fingerprint absorption peaks are present: 3,428 cm^−1^ (O-H stretching of -SO_3_H groups), 1,076 cm^−1^ (symmetric stretching of O = S = O), and 706 cm^−1^ (S-O stretching; Li et al., 2014b; Ma et al., 2018). For composite membranes, the intensity of the peaks for -SO${}_{3}^{-}$ (1,076, 1,020, and 706 cm^−1^) increases with the DS, because of the increased amount of the -SO_3_H. More importantly, all of the composite membranes exhibit the characteristic peaks of lignin, indicating the incorporation of lignin into the SPEEK matrix. Compared with the pure SPEEK, the peaks of O-H stretching (3,248 cm^−1^) shift toward the low frequency and the peak intensity decreases in the composite membranes. This suggests the hydrogen bond interaction between the -OH groups of lignin and the -SO_3_H groups of SPEEK.

**Figure 4:**
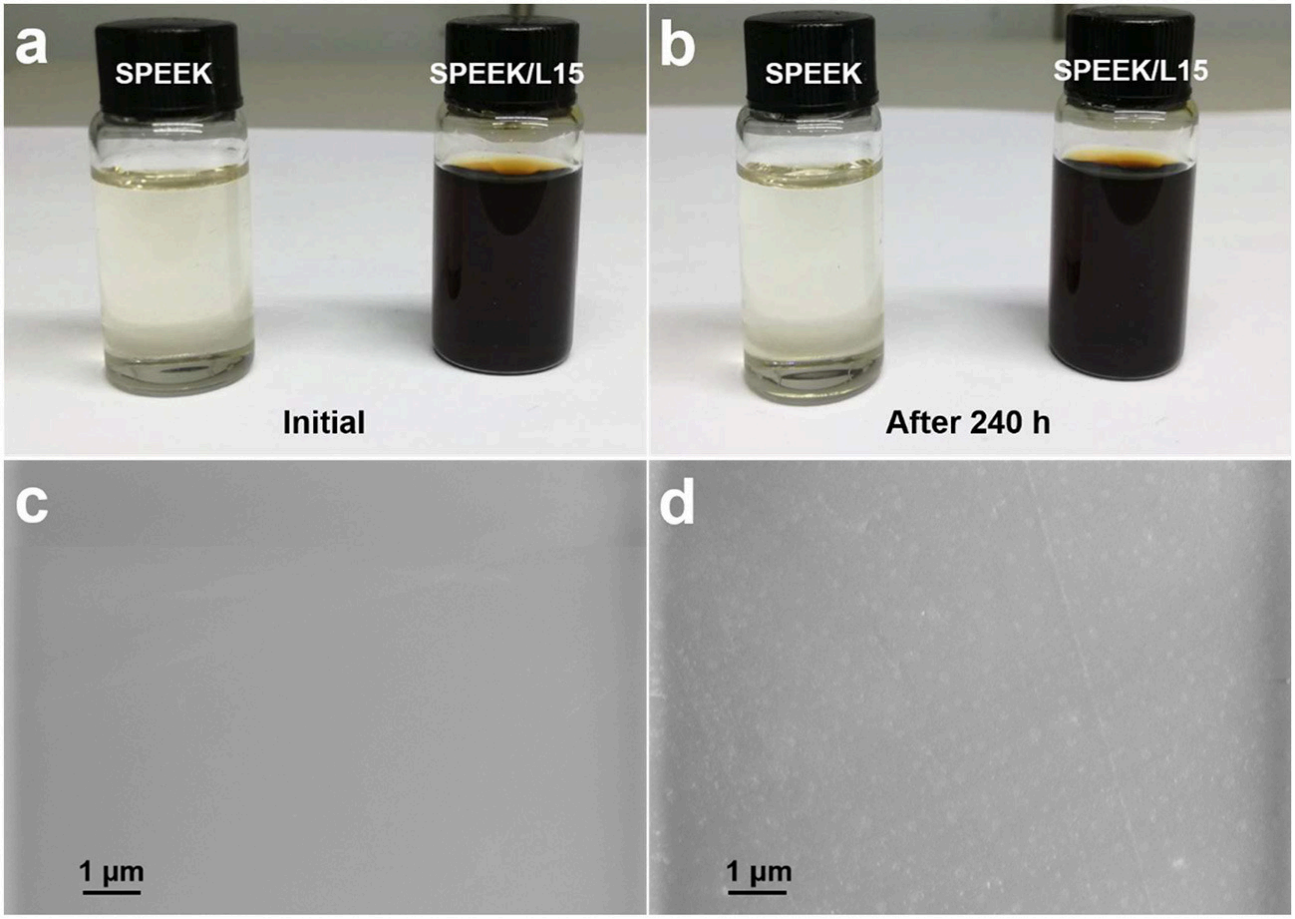
Photographs of SPEEK solution and SPEEK/lignin mixture in DMSO: **(A)** initial and **(B)** after 240 h; SEM images of **(C)** pure SPEEK and **(D)** SPEEK50/L15 membranes.

Figures 4A,B reveals that the lignin powders are homogeneously dispersed in the SPEEK solution without any precipitates, even after 240 h. This is a prerequisite for forming a uniform composite membrane. Figures 4C,D displays the resulting SPEEK and SPEEK50/L15 membranes which exhibit uniform and dense surface. This would hamper the crossing of vanadium ions and enhance the cycle stability of the membrane. Moreover, the lignin particles are homogeneously embedded into the polymer matrix and can improve the wetting property of the composite membrane. The 3D structure of lignin also provides more pathways for protons transport and reduces the water channels to repulse vanadium ions. Therefore, the lignin is introduced into the SPEEK matrix, which can enhance the proton conductivity while inhibit the vanadium ions permeation.

The proton transport generally proceeds via the vehicle and Grotthuss mechanism in the membrane. As such, the water uptake is a critical property. It has been demonstrated that the proton conductivity of the proton exchange membrane enhances with the WU amount. However, a high water uptake usually results in low mechanical stability. Table 1 shows that the WU of SPEEK/lignin membranes increases with the DS, as more hydrophilic -SO_3_H groups enhance the wetting property of the membrane. Similarly, the SR displays the same tendency as the WU.

**Table 1:** Property of different membranes.

**Membrane**	**Thickness (μm)**	**Area resistance (Ω cm^2^)**	**Water Uptake (%)**	**Swelling ratio (%)**	**Conductivity (mS cm^−1^)**
SPEEK41/L15	81	1.054	23.62	6.60	7.7
SPEEK50/L15	81	0.609	27.61	8.25	13.3
SPEEK59/L15	81	0.460	30.07	10.28	17.6
SPEEK72/L15	81	0.406	42.63	12.47	19.9
Nafion 212	50	0.244	/	/	20.4

**Figure 5:**
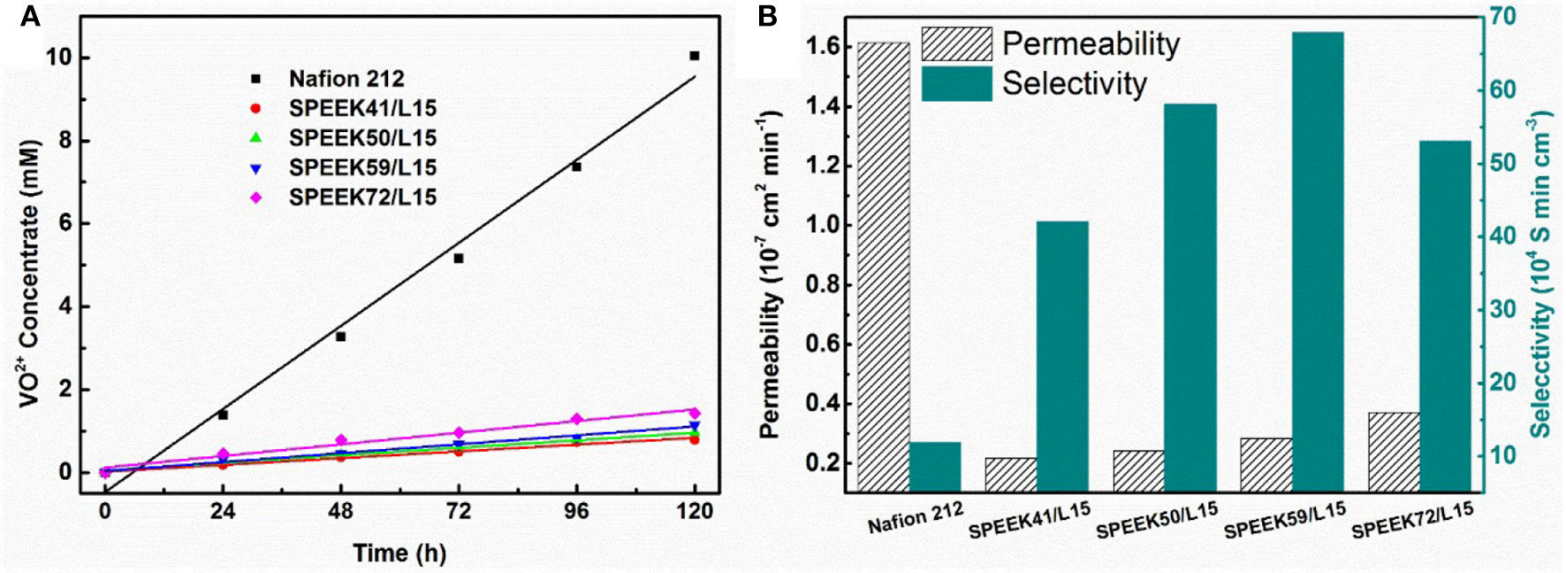
Comparison between Nafion 212 and SPEEK/Lignin membranes: **(A)** VO^2+^ permeability; **(B)** permeability and ion selectivity.

The permeability of the resulting membranes is shown in Figure 5. The VO^2+^ permeability of SPEEK/lignin membranes increases with the degree of sulfonation. This is attributed to the high DS that generally imparts abundant -SO${}_{3}^{-}$ groups in the polymer matrix, which drastically improve the proton conductivity (see Table 1) and also accelerate the crossover of vanadium ions through the membrane. The ion selectivity (Zhang et al., 2014a; Ji et al., 2017), namely the ratio of proton conductivity to ion permeability, is widely used to describe the balance between the two processes. Figure 5B shows that the ion selectivity of the composite membranes is much higher than that of the Nafion 212, regardless of the DS in SPEEK, because of the suppressed permeability of VO^2+^ ion. This clearly demonstrates the advances of the composite system. In particular, the SPEEK59/L15 membrane exhibits the best performance (61.96 × 10^4^ S min cm^−3^), nearly five-fold increment as compared to the Nafion 212 (12.78 × 10^4^ S min cm^−3^). The incorporation of lignin into the SPEEK substrate provides more proton transport pathway and meanwhile adjusts the water channels to repulse vanadium ions, thus giving rise to high ion selectivity.

### Cell performances

**Figure 6:**
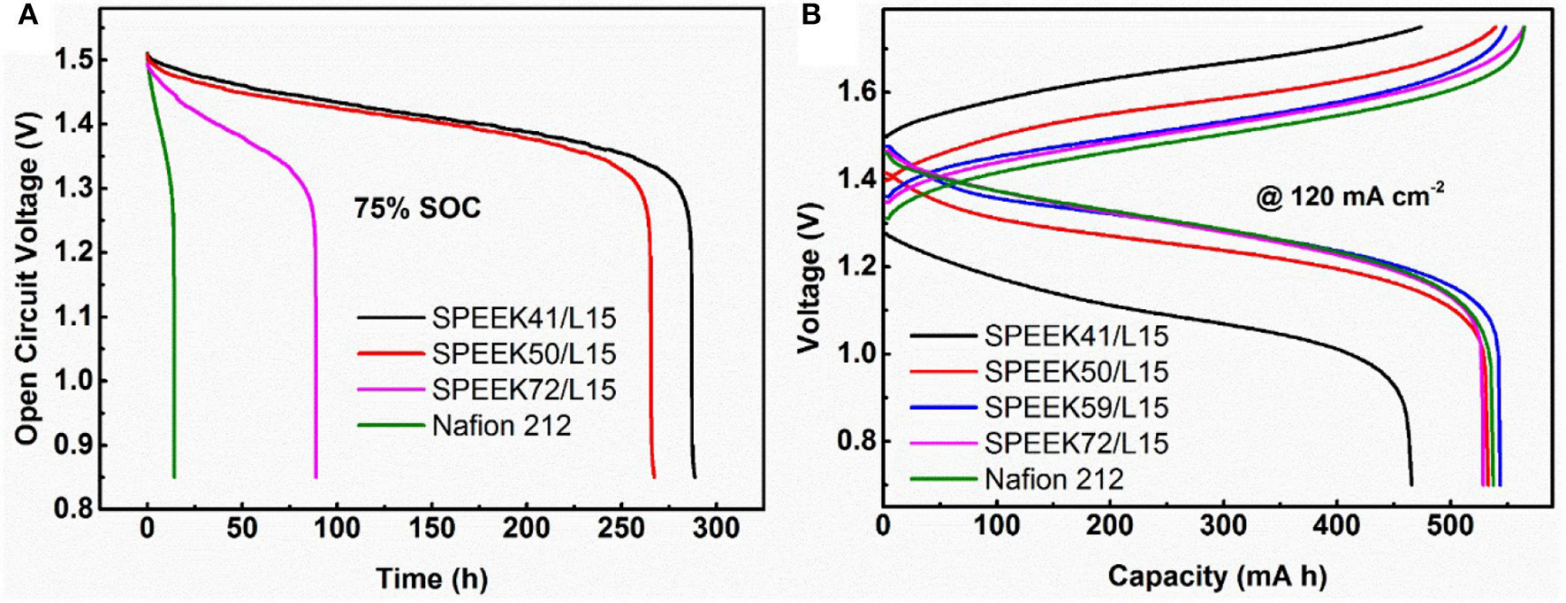
**(A)** Open circuit voltage decay of different membranes, **(B)** the charge-discharge performance of VRB cells employing different membranes at 120 mA cm^−2^.

To systematically study the performance of the composite membranes, the OCV, long cycle process, and rate performance of VRB single cells were carried out under different conditions. The OCV is a critical parameter to verify the vanadium ions cross rate in the membranes, as the vanadium ions crossing the membrane results in self-discharge and therefore the cell voltage declines accordingly. Figure 6A reveals that the OCV curves decrease rapidly with the enhanced DS in the composite membrane. It is obvious that the voltage decay (above 0.85 V) of cells assembled with the composite membranes is much slower than that of Nafion 212 (288.6 vs. 14.05 h for SPEEK41/L15 vs. Nafion). This indicates that the SPEEK/lignin composite membrane efficiently suppresses the permeation of vanadium ions, in good agreement with the results in Figure 5.

Figure 6B displays the typical charge-discharge curves of the VRB cells using different membranes under current density of 120 mA cm^−2^. As a high area resistance (see Table 1) usually leads to high ohmic polarization, the average charge voltage of cells with SPEEK/lignin membranes is slightly higher than that of the Nafion 212, with the average discharge voltage being on the contrary. However, the SPEEK59/L15 membrane exhibits the best discharge capacity, as a result of high ion selectivity, consistent with the above discussion.

**Figure 7:**
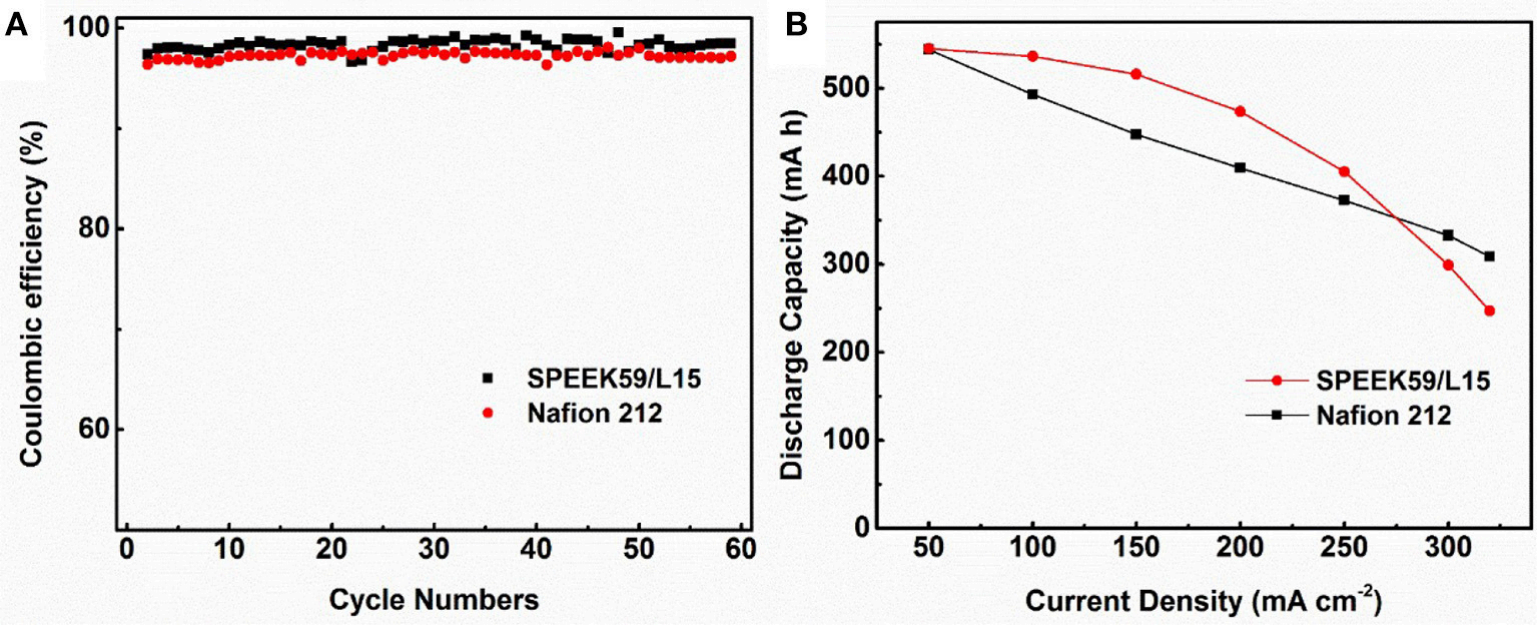
The Coulombic efficiency **(A)** and discharge capacity **(B)** of the cells with SPEEK59/L15 and Nafion 212 membranes at current density between 50 and 320 mA cm^−2^.

Figure 7 shows the cycling performance of cells assembled with SPEEK59/L15 and Nafion 212 membranes from 50 to 320 mA cm^−2^. The Coulombic efficiency of the cells with SPEEK59/L15 (up to 99.56%) is higher than that of Nafion 212 over the whole rate range. This originates from the reduced vanadium ion permeability with the composite membranes. It is noted that the CE of both cells increases with the current density. This is mainly due to the shortened charge-discharge time under high current density, which suppresses the crossover of vanadium ions through the membrane. Figure 7B reveals that the discharge capacity of SPEEK59/L15 is higher than that of the Nafion 212 under current density ≤ 250 mA cm^−2^. This can be attributed to the overpotential and ohmic polarization under high current. Therefore, the better performance of SPEEK59/L15 is ascribed to the synergistic effect from the lignin additives and SPEEK matrix, which provides more proton transport pathway and meanwhile suppresses the crossover of the vanadium ions.

**Figure 8:**
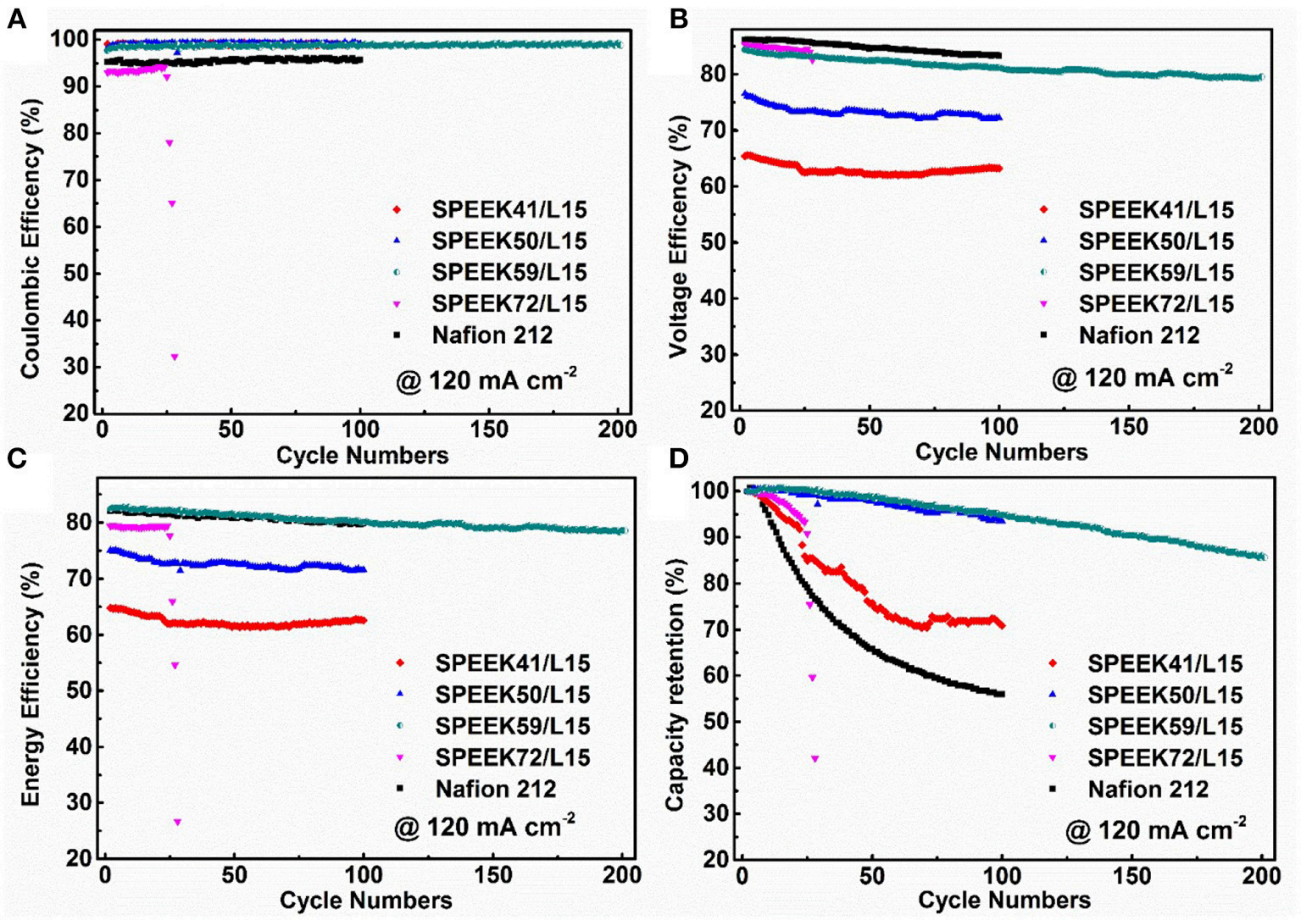
The Coulombic efficiency **(A)**, voltage efficiency **(B)**, energy efficiency **(C)**, and discharge capacity retention **(D)** of VRB cells using different membranes under current density of 120 mA cm^−2^.

Figure 8 displays the stability performance of the corresponding VRB cells operated at 120 mA cm^−2^. The Coulombic efficiency decreases with the degree of sulfonation (Figure 8A). This agrees well with the varying trend of VO^2+^ permeability (Figure 5A), as a high DS improves the proton conductivity but accelerates the crossover of vanadium ions through the membrane. As such, the voltage efficiency increases with the DS (Figure 8B). Accordingly, the cells with SPEEK59/L15 membranes exhibit an impressive energy efficiency (EE = CE × VE, up to 82.75%), comparable to that of the Nafion 212. This is attributed to the best ion selectivity for the SPEEK59/L15 membrane. It is noteworthy that the cells with SPEEK72/L15 fail after only 29th cycles, as a high DS deteriorates the mechanical and chemical stability of the membrane.

The capacity retention of VRB cells is a key factor to measure the ion imbalance in the operation process. Figure 8D discloses that the discharge capacity retention of the cells assembled with Nafion 212 maintains just 55.96% after 100 cycles under 120 mA cm^−2^. In contrast, under the same conditions, the cells with SPEEK59/L15 keep about 94.80% after 100 cycles, and more than 85% after 200 cycles. This demonstrates that the SPEEK59/L15 membrane substantially suppresses the crossover of vanadium ions and thus enhances the stability and prolongs the cycle life. The cells with the SPEEK59/L15 membranes show the best capacity retention and outperform the other ones. The SPEEK59/L15 membranes synthesized by an eco-friendly and cost-effective approach exhibits high ion selectivity and excellent stability, making it a promising candidate for efficient VRB system.

## Conclusions

The SPEEK/lignin composite membranes were optimized by controlling the degree of sulfonation toward the VRB application. The VRB cells with SPEEK59/L15 membranes exhibit an impressive energy efficiency up to 82.75%, low vanadium ion permeability, high ion selectivity, and high capacity retention (94.80% after 100 cycles and over 85% after 200 cycles). The good performance is assigned to the synergistic effect from the lignin additives and SPEEK matrix, which improves the proton conductivity and suppresses the crossover of the vanadium ions. The eco-friendly and cost-effective composite membranes make it a competent candidate for VRB energy storage technique.

## Author contributions

MD, LS, and CJ conceived the idea and supervised the work. JY and XL synthesized the membranes and performed most of the experiments. JY, XL, CW, and SW prepared the samples and analyzed the data. JY, XL, MD, LS, and CJ participated in analyzing the data and wrote the report. All authors read and approved the final manuscript.

### Conflict of interest statement

The authors declare that the research was conducted in the absence of any commercial or financial relationships that could be construed as a potential conflict of interest.
